# Reasoning Talk at Chinese Families’ Dinner Table: Across Three Generations and Different Communicative Contexts

**DOI:** 10.3389/fpsyg.2021.763625

**Published:** 2021-12-02

**Authors:** Lifang Liu, Feiyi Zheng, Ling Sheng, Yijun Hao, Jiangbo Hu

**Affiliations:** Hangzhou College of Preschool Education, Zhejiang Normal University, Hangzhou, China

**Keywords:** reasoning talk, three generations, communicative contexts, mealtime talk, Chinese families

## Abstract

This study examines the feature of reasoning talk used by 37 Chinese families at the dinner table across three generations with the background of co-parenting and in consideration of different communicative contexts. Drawing upon Hasan’s semantic framework, reasons were mainly coded as logical or social types. We categorize the communicative context of reasoning talk into contextualized (meal-related) and decontextualized topics. When the proportion of social reasoning was found slightly higher than that of logical reasoning, the families’ reasoning talk account for only 3.85% of the total language. Specifically, the count of mothers’ total reasoning talk was significantly above other family members, while there were no significant differences among the other participants. The effect of the communicative contexts on family members’ social reasoning was found. The reasoning talk grounded on local rules (family-made rules) and coercive power occurred significantly more frequently in contextualized than decontextualized context. A higher rate of local-rule grounded reasoning talk of all family members appeared in contextualized than decontextualized context, and this gap was particularly obvious among mothers. These findings reveal the significant role of mothers in family communications and confirm the pedagogical values of decontextualized communicative context for promoting children’s learning opportunities at the dinner table.

## Introduction

Reasoning talk, identified as a type of language containing causality, is associated with a child’s development in social cognitive abilities (Brumark, 2006; Frazier et al., 2009; Hasan, 2009; Hu and Torr, 2016; Hu et al., 2019a). If children often engage in reasoning talk with adults that comprise logical links based on the law of nature, their logical thinking can be invisibly shaped, and they will gain more experience in scientific conception that the school attaches importance to Callanan and Oakes (1992), Kelemen et al. (2005). A wealth of research demonstrates the significance of parents’ use of reasoning talk in their daily conversations with children, yet much of the current knowledge is derived from research in Western cultures and few studies focus on reasoning talk in Chinese families. This study attempts to examine the manners that Chinese parents and children are engaged in reasoning talk in the context of the dinner table, a fixed family routine that involves all family members who generate conversations spontaneously.

Research suggests that dinner table talk constructs multiple interactive contexts where the richness and complexity of conversational language would increase, and this is viewed as a significant language learning context for children (Bohanek et al., 2009; Fruh et al., 2011; Hu et al., 2019b). Research finds that certain genres of parent-child mealtime talk including explanatory, narrative, and justification discourse that relate to decontextualized topics positively associated with children’s expressive language and social-cognitive skills (Aukrust, 2002; Brumark, 2006; Snow and Beals, 2006; Bova, 2011; Rowe, 2013; Bova and Arcidiacono, 2014). Reasoning talk as a type of language explaining causal relationships is frequently used in these genres for explaining or justifying speakers’ opinions on various topics. The dinner table can be an ideal context for the exploration of families’ reasoning talk.

Unlike the existing research that mainly focuses on parent-child talk, this study investigates reasoning talk across three generations including parents (mothers and fathers), grandparents, and children. The co-parenting pattern in Chinese families where grandparents live in their children’s home to share childcare burdens is popular (Jiang et al., 2007; Wang et al., 2019). Grandparents play an important role in Chinese young children’s lives, and this is especially the case in the context of dinner tables where grandparents are regular participants. This study is designed to investigate the reasoning talk during dinner time involving parents, grandparents, and children, which is rarely reported in the literature of both dinner table talk and reasoning discourses. The findings would increase our understanding about reasoning talk spontaneously occurring in Chinese families that have an impact on Chinese young children’s language learning, logical thinking, and social understanding.

## Reasoning Talk and Child Development

The literature represents reasoning talk as science talk, causal talk, or explanatory talk that contains the explanation of cause-and-effect relationships. Studies show that there exists an association between parent-child reasoning conversations and children’s cognitive development that has implications for their future academic success in school (Callanan and Oakes, 1992; Kelemen et al., 2005; Hasan, 2009; Booth et al., 2020). For example, Booth et al. (2020) observed 153 parent-child dyads in a museum and a lab and found that parents’ initiation of engaging children in science talk is positively related to children’s performance of scientific literacy in schools. In reasoning conversations, children could incorporate their parents’ interpretation of affairs into their developing representations of different categories. Therefore, the reasoning dialogue that young children hear and participate in daily life might be the precursor of their cognitive and linguistic needs in dealing with explanatory texts and conversations in schooling life (Hasan, 2009). Parents’ reasoning talk enriches the sources of young children’s information learning and enhances their understanding of the surrounding world.

The factors relating to parents’ socio-cultural background can impact parents’ reasoning styles. Hasan (2009) compared reasoning talk of professional (well-educated) and non-professional mothers in Australia. The results showed that professional mothers employed more reasoning talk grounded on logical laws than the non-professional mothers when directing children’s behaviors or explaining phenomena in daily life. The mothers of non-professional backgrounds tended to use social reasoning involving personal authority or coercive power to direct children’s behaviors, such as “If you don’t listen, you will not go to bed with Teddy.” This finding is partially in line with Tenenbaum and Callanan (2008) who investigated parent-child science talk of higher schooling groups (completed secondary school) and basic schooling groups (fewer than 12 years) at the museum and the home. In both contexts, the parents of the higher schooling group used more scientific-oriented causal explanations than basic schooling group parents in their conversations with children. A particularly relevant study undertaken by Hu and Torr (2016) investigated the nature of five Australian Chinese mothers’ (university educated) reasoning talk with their children in daily conversations. This study reveals that Chinese mothers generally used more reasoning talk grounded on social rules than logical reasoning drawn on natural laws. Among social reasoning, the Chinese mothers mostly used “cooperative reasoning” (e.g., you cannot take that car because it is your brother’s favorite), which was underpinned by the value of maintaining harmonious relationships among people that is rooted in Chinese culture.

Communicative context is another factor that could impact the adults’ use of reasoning talk. Hu and Torr (2016) found that regulatory topics (relating to children’s behaviors) triggered more social reasoning, whilst non-regulatory topics fostered more logical reasoning in Chinese mothers’ child-addressed language. Another study resonates with this finding, which investigated Australian educators’ reasoning talk with infants. The researchers found that whilst educators of university qualification generally used more reasoning talk than the educators of lower qualification, logical reasoning occurred more frequently than social reasoning in non-regulatory context regardless of different qualified educators (Hu et al., 2019a). These findings suggest that the influences of communicative contexts on reasoning types may exist in both educational setting and home environment.

## Dinner Table Talk and Children’s Language Learning

Dinner table talk is a supportive context for the use of language including rare words and complex syntax which potentially create extensive language learning opportunities for children (Snow and Beals, 2006; Sheng et al., 2021). The pedagogical functions embedded in dinner table talk could be more than toy play or even shared reading (Weizman and Snow, 2001). Snow and Beals (2006) analyzed different types of language that parents produced during mealtime and identified the pedagogical values of parents’ language. The frequency of parents’ use of extended discourses (explanatory or narrative talk) and rare words at the dinner table is positively associated with children’s vocabulary and language expression skills. Hu et al. (2019b) explored five Chinese Australian mothers’ language use during mealtime from the perspective of interpersonal functions (offer, demand, question, and statement). It reveals that the parents’ choices of different interpersonal functions in their conversations with children would spontaneously shape children’s communication roles (e.g., demand complier or question answerer), which may impact children’s language skills subtly in terms of role-playing in communications.

Language learning opportunities of mealtime talk may vary according to topics. Some topics are contextualized that focus on food or children’s behavioral management (here-and-now), whereas some are decontextualized and related to affairs beyond the dinner table such as social events or past experiences (there-and-then). Research suggests that decontextualized topics may trigger extended genres, such as explanatory or narrative talk, which contain sophisticated vocabulary or complex syntactic structure that predict children’s future language and literacy skills (Dickinson and Tabors, 2001; Snow and Beals, 2006). In comparison, contextualized conversations involve more directive language for addressing food eating or children’s table manners, which may restrict children’s language learning opportunities (Sheng et al., 2021).

Apparently, little research directly addresses reasoning talk during mealtime, however, the research exploring argumentation involving reasoning discourses gives a clue in this field. Research suggests that parent-child arguments provide opportunities for children to practice logical thinking and prepare for contests involving the consideration of the other side’s psychological states (Arcidiacono and Bova, 2015). Parents generally dominate the dinner table debate. Through the investigation of the content of the parent-child debate, Bova and Arcidiacono (2014) summarized a few reasoning strategies that parents applied mostly for the justification at the dinner table, including “appeal to food quality and quantity” (you need to eat more vegetables because it is nutritious), “appeal to consistency” (you had two serves of rice yesterday, so you can have two serves today as well), and “appeal to authority” (Your teacher said tomorrow is a pajama day, so we need to choose a pajama tonight). Children usually play a subordinate role at the dinner table arguments as children’s reasoning skills are weaker than their parents’. Unlike parents who employ a variety of strategies, young children (preschool-aged) mainly express their doubts and cannot initiate effective arguments (Bova and Arcidiacono, 2014), though some preschoolers can challenge parents with “Why” questions to force parents trying to verify their rules with reasons (Bova, 2011). Compared to older children (teenagers), the reasoning strategies of children under 10 years are more likely connected with the here-and-now context that only focuses on one or two concepts (Brumark, 2006; Arcidiacono and Bova, 2015).

## Parent-Child and Grandparent-Child Interactions in Chinese Families

With the increase of social competition pressure in China, many parents prefer to share the duties of childcare with grandparents. That is, there is a tendency for Chinese parents to raise their children together with their grandparents. According to the study of Xu and Pei (2012) who selected 301 children’s like the research objects through cluster sampling and surveyed their caregiving situations, the results showed that 66.5% of these children’s families had grandparents living together with them, and most grandparents participated in these children’s daily caring activities. In the families where three generations live together, grandparents are important family members who play a crucial role in creating the family environment that may impact the cognitive and social development of children (Denham and Smith, 1989; Xu and Pei, 2012; Gao et al., 2016). However, relevant research shows mixed results about the influence of grandparents in families. On one hand, it finds that grandparents’ participation in children’s everyday activities could effectively improve parents’ self-efficacy on child-rearing routines and promote family harmonious relationships which benefit children’s prosocial behavior (Xu and Pei, 2012; Gao et al., 2016). While on the other hand, grandparents can be overprotective and are more likely to use authoritarian ways to direct children’s behaviors in their interactions with children (Lin, 2009). This is especially the case when grandparent interacts with children during mealtimes regarding children’s eating issues, during which grandparents particularly pay attention to the quantity of the food that children intake.

Differing from grandparent’s interactional styles, Chinese parents pay more attention to children’s educational issues and actively engage in literacy (e.g., shared reading) and numeracy activities with children (Zhang and Koda, 2011; Hsu, 2015). Young generations of Chinese parents are reported to embrace warm and open communication in their interactions with children, showing respect to children’s autonomy as independent communicators (Hu et al., 2014; Hu and Torr, 2016). In an investigation of Chinese parents’ language attitudes and practices relating to their preschool-aged children’s language development, some parents showed their preference to interact with their children in an easy and play-based approach, which is different from the experiences they had when they were a child. A mother in the investigation expressed the idea regarding her interactions with children during educational activities, “When we were young, we were forced by our parent to do things…., but I want them (her daughters) to learn happily and learn through playing” (Hu et al., 2014, p. 149). Fathers and mothers may differ in their interactions with children as well. In general, mothers spend more time than fathers in accompanying and interacting with their children in Chinese families, though fathers have been increasing their interactions with children in the recent decade (2008–2017) and are establishing warmer relationships with children (Du et al., 2018). Compared to the mothers who interacted with children with multi-faced styles, Chinese fathers tend to play a mentor role and give children advice (Li, 2020).

It should be noted that although the existing studies revealed the nature of caregiving in the Chinese families of three generations living together, most of such studies focused on parents’ or grandparents’ individual interactions with children rather than their shared parenting in one situation. This study is designed to exam how three generations of Chinese families use reasoning talk (a specific language type) at the dinner table, which may have substantial implications to the children’s learning opportunities relating to their development in language, cognitive, and social domains.

## A Theoretical Framework for Analyzing Reasoning Talk

This study draws on systemic functional linguistic theory, specifically Hasan (2009) theory of reasoning talk for the analysis of the language generated by the Chinese family members at the dinner table. Grounded on semantic meaning of language, Hasan identifies reasoning talk with four steps’ reasoning chain that includes, (a) claim, that can be presented as a statement, offer, command or question; (b) reason, that shows a cause-and-effect relationship in the language; (c) principle, that validates the reason with a generalized notion; (d) grounding, that serves as the fundamental endorsement of the principle. For example, in a sentence of “We will have rice cakes after the dinner because it is the Dragon Boat Festival,” the *claim* is the statement of *we will have rice cake* (claiming message), the *reason* is *it is the Dragon Boat Festival* (reasoning message), the *principle* is the convention of eating rice cake in Chinese Dragon Boat Festival, and the *grounding* belongs to the type of “communal” that indicates the principle shared by Chinese communities. According to Hasan (2009), principle and grounding are always implicit in the speakers’ language, however, they exist and determine the validity of the reasoning talk.

Hasan (2009) argued that grounding is the fundamental endorsement of the whole reasoning chain, and based on this notion, she categorized reasoning talk according to the types of grounding. The two basic types of grounding are logical and social. The logical grounding has unquestionable authority determined by natural laws, for example, *we need to put sunscreen on such a hot day otherwise our skin may get sunburn*. This statement is grounded on the natural law that direct exposure to sunlight may lead to sunburn on a hot day. The natural laws in the physical world appear to be numerous and complex, but they are all “nature-made” principles and Hasan categorized them as one type. In contrast, social rules created by human society hold different views on social events. Social reasoning based on social rules are changeable and often can be challenged or questioned, representing the values of different groups or institutions, such as you *cannot go out without the uniform as it is the requirement of the school* (this is a rule created by people and can be changed).

There are two dimensions in the classification of social reasoning. The first dimension considers who makes the rule. According to the rule-makers, three subcategories can be identified for social grounding: local (small group, highlighting individual authority), communal (social group, directing social forces), and institutional (the authoritative group, representing mandatory and legal binding force). The other dimension defines two subcategories according to the relationships between the participants in the interaction, which include cooperative (showing the equality and mutual benefit) and coercive (suggesting the inequality and the burden of one side on the other). The examples of the six types of reasoning grounding are summarized in Table 1.

**TABLE 1:** Reasoning types based on the six groundings.

**Reasoning types**		**Examples**
Logical		You need to put on your gumboots later. It’s very wet outside.
Social- based on rule-makers	Institutional	There are so many things you cannot do in this theme park as today is a holiday.
	Communal	A princess also eats rice dumplings today because it is Dragon Boat Festival.
	Local	If you are the last one to finish the dinner, then you should wash the dishes.
Social- based on relationships between participants	Cooperative Coercive (threat, bribe, and emotional blackmail)	Your sister is sleeping. We speak in small voice. If you finish the meal properly, then you can have an ice cream soon (bribe).

The types of grounding reflect speakers’ internalized views on the relationships of affairs in the world that are either logical or social. The reasoning manner that parents employ habitually in their daily conversations with children passes their ideas, attitudes, and understanding about the social and natural world to children effortlessly and instinctively. From this point of view, parents’ reasoning talk would essentially impact children’s social and cognitive understanding as an “idea-input channel.”

## Research Aims

Research has established the pedagogical functions of reasoning talk and mealtime talk that have critical implications for children’s language learning and social and cognitive development. Chinese grandparents play an important role in child-rearing activities and contribute to a “co-parenting” style in Chinese families. The present study aims to explore the feature of reasoning talk at Chinese families’ dinner tables across the three generations. This study contributes to the relevant literature in two aspects. Firstly, it enriches the research field of mealtime talk with Chinese families’ data and focuses on reasoning talk that was rarely explored. Secondly, it differs from most research on Chinese children’s home experiences that draws on investigations on parents’ attitudes and practices through methods of interviews or questionnaires. The present study employs corpus study to analyze the text of language produced by Chinese family members spontaneously, which is methodologically innovative in research. In particular, this study aims to address the following two questions.

• What are the frequency and types of reasoning talk generated by Chinese parents (mother and father), grandparents, and children at the dinner table?
• What are the characteristics (types) of reasoning talk of Chinese parents (mother and father), grandparents, and children in different communicative contexts (contextualized and decontextualized)?

## Materials and Methods

### Participants

This study is a part of a larger project of Chinese Children’s Early Language Experiences (CCELE) that investigates Chinese young children’s language experiences in all sorts of social interactions with other people (educators, family members, and peers) that determine the language environments of children. For this study, the participating families were all from one state-owned preschool in [Deqing, Zhejiang Province], an economically advanced small city in China. As the main objective of this study is to reveal the nature of reasoning talk in Chinese families across three generations rather than across different socioeconomic groups, we control for the variation of the families’ background. The criteria of the participating family selection include, (i) the families adopt co-parenting pattern and have three generations who have dinner together regularly; (ii) the families have one single child attending preschool; (iii) the grandparents, parents, and the children are at normal levels of intellectual and language development, or in other words, they are mentally and physically healthy; (iv) one of the parents is university educated and has a professional background. This selection represents the fast-increasing middle-class families in Chinese small cities, which is also a fast-growing group in the Chinese population (Chinese National Bureau of Statistics, 2021). Among the participating parents, 78% of the mothers and 81% the fathers obtained university degrees, whilst none of the participating grandparents was reported having tertiary educational experiences. The participating children’s age varied from 3 to 6 years (*Mean* [*M*] = 52 months).

One noticeable point is that more than half (57%) of the participating families only had one grandparent in the household while the others had two grandparents. To ensure the comparability among the families, we compared grandparents’ prevalence of reasoning talk between the families with one and two grandparents. According to the Independent Samples *t*-test, the differences between the two groups were not significant (*t* = 1.25, *p* > 0.05). Therefore, we combined the two categories and did not separate grandmother and grandfather as different family members like mother and father.

### Data Collection

Digital video record was used as the main data collection method in this study. To reduce the influence of observing acts from researchers on the naturalistic data collection, family members were invited as participant observers to videotape one family dinner event at their convenient time. The ethical approval to undertake the study was obtained from the Human Ethics Committee of [Zhejiang Normal University]. The consent was obtained from the participating parents who were aware of the requirement about the videotaping (e.g., setting the camera/smartphone to capture the whole context of dinner table interactions). The parents were advised to discuss this event with grandparents and children before handing in the consent. The parents took the responsibility to record the instances of their dinner table interaction as suggested. The video clips collected from the families varied from 17 to 30 min approximately. All the video records were transcribed verbatim.

### Coding Scheme

#### Messages

The transcribed data was broken into messages. A massage is a semantic unit that approximately equals to a cause that comprises one subject (explicate or implicate) and one verb (Hasan, 1996). A sentence may contain more than one message, such as *you filled a bowl of rice yourself and so you need to finish it your own*, which includes two massages: *you filled a bowl of rice/and so you need to finish it your own*. Body language that contains clear semantic meaning in interactions was also marked in the transcription, such as “waving hands” to indicate a rejection, which stands as an independent message.

#### Identification of Reasoning Talk

The broken messages were then reviewed to decide whether there is a cause-and-effect relationship semantically. The number of reasoning talks was counted according to the occurrence of reasoning messages, the second step on the reasoning chain based on Hasan (2009) theoretical framework. Claiming messages, the first step on the reasoning chain was excluded though they appeared in the participants’ language because it is *reason* rather than *claim* that are associated with the grounding types. Extract 1 displays the identification of reasoning messages from total messages.

Extract 1: (The child started to laugh while eating food)
Mother: Don’t laugh.
Mother: You could be chocked **(reasoning message, logical)**
Mother: Smart children don’t laugh when having meals **(reasoning message, local)**
Child: (Swallowed the food and stopped laughing).

#### Categorization of Reasoning Types

Each reasoning message was categorized according to the types of grounding that can be generally divided into logical or social grounding as stated before. For social reasoning, we further sorted the messages into sub-categories.

#### Initiator of Reasoning Message

Any family member including grandparent, father, mother and the child might provide reasons to justify his/her own or another family member’s claim. For example, a grandmother said to the child “You need to have some vegetables,” and the mother provided the reason “yes, it is nutritious.” In this situation, the initiator of the reasoning message is the mother.

#### Communicative Context

The communicative context of family reasoning was coded into contextualized and decontextualized topics. Contextualized topics are in relation to the meal, such as the taste of the food and/or the behaviors of family members at the table. Decontextualized topics refer to the information that belongs to “there-and-then” issues, for example, *this kind of fish cannot be found in the Chinese Eastern Sea, it must be imported from overseas*.

### Inter-Coder Reliability

To check the reliability of the coding, one researcher coded the total data of the 37 families based on the above coding scheme independently, which was followed by another researcher who randomly chose and recoded eight cases from the 37 cohorts. The rate (22%) of recoded cases meets the standard for establishing reliability for coding narrative data (Syed and Nelson, 2015). Cohen’s kappa coefficiency was calculated for all the variables: the coefficients were 93% for the reasoning messages, 89% for the reasoning types, 100% for the initiators (family members), and 84% for the type of communicative context.

### Data Analysis

Data analysis was undertaken according to the two research questions. To address the first research question, the descriptive analysis of the reasoning talk (reasoning messages) by different family members was presented. To address the second question, a 4 (family members including mother, father, grandparent, and children) × 2 (contextualized and decontextualized communicative context) MANOVA was used to explore the differences of reasoning messages generated by the different family members under different contexts, with the types of reasoning talk as dependent variables. As the duration of dinner time varied across the families, the variable of reasoning talk is represented by the proportion rather than the number of reasoning messages. The proportion of reasoning talk was calculated by dividing the total reasoning messages by the total messages of each family. The proportion of reasoning type was calculated by dividing each type of reasoning message by the total messages that each family generated. The data analyses were carried out in IBM SPSS 23, United States.

## Results

### Descriptive Statistical Analysis on the Use of Reasoning Talk by Three Generations of the Family Members

Table 2 presents the descriptive statistics of the proportion of total and six types of reasoning talk by different family members. In total, there were 394 reasoning messages identified from 10,313 messages produced by the 37 families at the dinner table. Reasoning talk takes up 3.85% of the overall language with the standard deviation at 1.63%. This finding suggests that reasoning talk is not a frequently used language type in these families, and significant differences exist among the families. According to the observational data, most of the language used by the family members were statements, commands, offers, and questions without the supplement of reasoning messages, such as “The fish tastes delicious, where did you buy them? Here you are, you are welcome to have more.”

**TABLE 2:** Descriptive statistics of the proportion of reasoning talk generated by the participating family members.

**%**	**Mother**		**Father**		**Grandparent**		**Children**		**Family**	
	***M***	***SD***	***M***	***SD***	***M***	***SD***	***M***	***SD***	***M***	***SD***
total messages	38.44	13.70	18.22	11.99	18.72	12.16	24.61	7.39	/	/
Reasoning Talk	1.77	1.45	0.83	0.77	0.55	0.66	0.71	0.75	3.85	1.63
Logical	0.87	0.78	0.28	0.36	0.24	0.34	0.31	0.43	1.70	1.00
Social	0.90	0.92	0.55	0.63	0.32	0.53	0.40	0.49	2.16	1.33
Cooperative	0.02	0.12	0.03	0.12	0.01	0.06	0.03	0.10	0.09	0.19
Institutional	0.03	0.13	0.02	0.07	0	0	0	0	0.05	0.14
Communal	0.17	0.29	0.06	0.22	0.05	0.13	0.02	0.07	0.30	0.42
Local	0.57	0.86	0.29	0.39	0.20	0.34	0.33	0.41	1.39	1.13
Coercive	0.10	0.18	0.15	0.40	0.06	0.19	0.02	0.11	0.33	0.60

In terms of the categories of reasoning talk, the proportion of social reasoning (2.16%) is slightly higher than logical reasoning (1.7%). Among the social grounded reasoning talk, the proportion of local grounding accounted highest, the mean is 1.39%. Coercive ranked second (0.33%), which is slightly higher than the type of Communal (0.30%). The proportion of cooperative and institution is minor, taking up 0.09 and 0.05%, respectively. As these two types of reasoning talk accounted for very small proportions, they were excluded in the follow-up analysis and discussion.

For the different family members, the mother played a significant role in these Chinese families’ dinner table. Mothers produced the highest rate of messages (*M* = 38.44%; *SD* = 13.70%) and reasoning talk (*M* = 1.77%; *SD* = 1.45%). Children were also very active in the mealtime, and they produced the second most messages (*M* = 24.61%; *SD* = 7.39%) and third ranked reasoning talk (*M* = 0.71%; *SD* = 0.75%). For the grandparent, they produced relatively more messages than the fathers during the mealtime talk (*M* = 18.72%; *SD* = 12.16%), however, the proportion of reasoning talk in grandparent’ language is the lowest (*M* = 0.55%; *SD* = 0.66%). At the family dinner table, the fathers produced the least messages (*M* = 18.22%; *SD* = 11.99%), yet the proportion of their reasoning talk is higher than the grandparents and children (*M* = 0.83%; *SD* = 0.77%).

### The Characteristics of Reasoning Types Across Three Generations and Different Communicative Contexts

To clarify the influences of family members (mother, father, grandparent, and children) and communicative contexts (contextualized and decontextualized) on the characteristics of reasoning types, we conducted a 4 × 2 MANOVA to examine the effect of these two factors (independent variables) on the proportions of reasoning types (dependent variables). The results are reported as follows.

#### The Effect of Family Members on Reasoning Types

**FIGURE 1:**
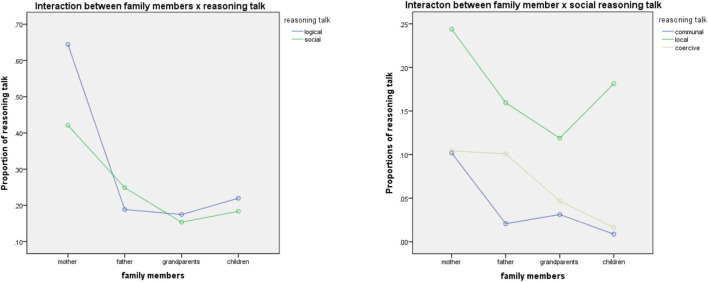
The interaction between family members × types of reasoning talk.

As shown in Figure 1, the results of the 4 × 2 MANOVA showed that the main effect of family members had a significant effect on reasoning talk [*F*(3,288) = 11.66, *p* < 0.01, partial η^2^ = 0.11], and the effect was shown in both logical [*F*(3,288) = 13.13, *p* < 0.01, partial η^2^ = 0.12] and social reasoning talk [*F*(3,288) = 4.79, *p* < 0.01, partial η^2^ = 0.05]. Among the three types of social reasoning (communal, local, and coercive), two of them showed significant differences among the family members, namely communal [*F*(3,288) = 4.04, *p* < 0.01, partial η^2^ = 0.04] and local [*F*(3,288) = 3.01, *p* < 0.05, partial η^2^ = 0.03].

The *post hoc* pairwise analysis revealed that mothers’ logical reasoning messages (*M* = 0.86%; *SD* = 1.06%) were significantly higher than fathers’ (*M* = 0.39%; *SD* = 0.57%), grandparents’ (*M* = 0.27%; *SD* = 0.51%) and children’s (*M* = 0.34%; *SD* = 0.50%), yet there were no significant differences among the other three participants. The difference of communal and local grounding indicated the same tendency, that is, mothers produced significant higher communal and local reasoning talk than the other three family members (communal: *M* = 0.09 > 0.03, 0.02, and 0.01%; local: *M* = 0.28 > 0.15, 0.10, and 0.17%). However, in relation to coercive reasoning talk, the fathers’ rate is considerably high, making up 0.75% of the families’ total language, which is higher than the mothers (0.52%) who produced most reasoning talk. This finding indicates that fathers used extensive individual authority for social justification at the dinner table.

#### The Effect of Communicative Contexts on Reasoning Types

**FIGURE 2:**
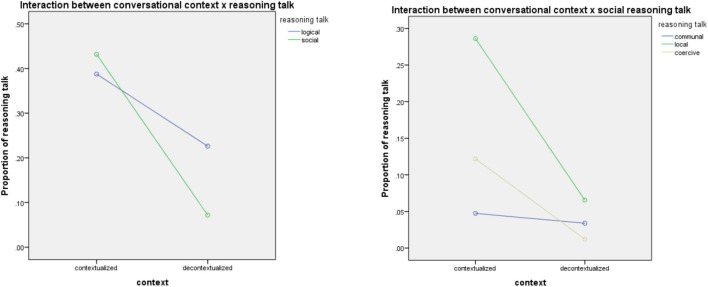
The interaction between communicative contexts × types of reasoning talk.

The main effect of communicative contexts had a significant effect on the social reasoning talk [*F*(1,288) = 24.05, *p* < 0.01, partial η^2^ = 0.08]. As Figure 2 depicted, only social reasoning talk had a significant difference between the two communicative contexts [*F*(1,288) = 43.35, *p* < 0.01, partial η^2^ = 0.13] whereas logical reasoning was not impacted by the communication contexts. Specifically, two out of the three types of social reasoning talk showed significant association with the contexts, namely local [*F*(1,288) = 33.10, *p* < 0.01, partial η^2^ = 0.10] and coercive [*F*(1,288) = 12.58, *p* < 0.01, partial η^2^ = 0.04]. The result of the descriptive analysis showed that reasoning talk grounded on local and coercive occurred significantly more frequently in contextualized (*M* = 0.30%, *SD* = 0.32%; *M* = 0.08%, *SD* = 0.15%) than in decontextualized context (*M* = 0.45%, *SD* = 0.03%; *M* = 0.04%, *SD* = 0.01%). In contextualized situations, such as talking about food intake and meal routines, the family members tended to use local rules (family rules) and individual authorities to justify their reasons.

#### The Interaction Effects of Family Members and Communicative Contexts on Reasoning Types

**FIGURE 3:**
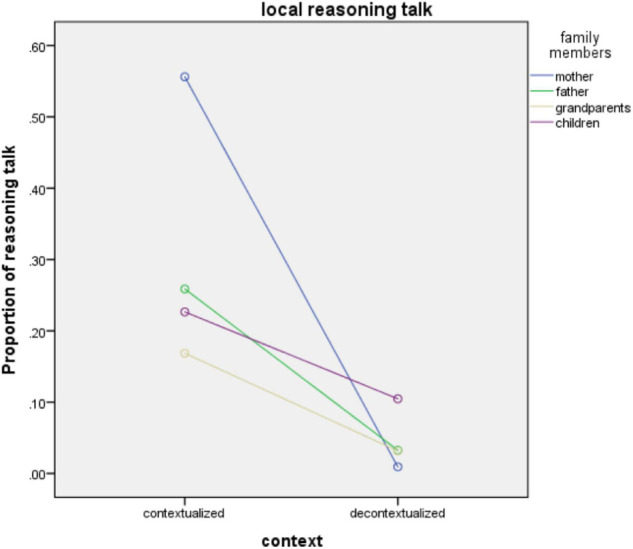
Family members use local reasoning talk in different communicative contexts.

For the interaction effects between family members x communicative context, the results showed a significant effect on the local reasoning talk [*F*(3,288) = 4.88, *p* < 0.05, partial η^2^ = 0.05]. This effect is displayed evidently in Figure 3. No significant interaction effects were found among other types of reasoning talk by different family members in different contexts. For the local reasoning talk, all the family members produced more local reasoning talk in contextualized than decontextualized conversational contexts. This gap was much more obvious among mothers who on average produced 0.56% contextualized local reasoning and 0.09% decontextualized local reasoning, respectively. The disparity among father and grandparents were also substantial (father: 0.26 vs. 0.03%; grandparents: 0.17 vs. 0.03%), though the difference among children is not significant (0.23 vs. 0.11%). Observational data shows that in this situation, the grandparents and both parents were more likely to use “homemade rules” (e.g., eat more vegetables, you will look pretty) to encourage children to eat more food.

## Discussion

### General Feature of Reasoning Talk in Different Communicative Contexts

This study explored the characteristics of reasoning talk among three generations at the dinner table in a group of middle-class Chinese families. It reveals the spontaneously occurring family reasoning talk in different communicative contexts across three generations, which increases our understanding of the learning opportunities that family mealtime can provide to children. As shown in this study, reasoning talk was not a frequently used language during mealtime talk through substantial variation existed among the cases. Most of the conversations at the families’ dinner table were expanded with family members’ agreement or extension on each other’s ideas. This result is partially in line with Sheng et al. (2021) recent study that reported the types of Chinese families’ mealtime conversations mostly without conflicts, and consequently, the opportunities for the family members to use reasoning discourse for explaining or defending self-opinions were limited. The cultural convention could be an important factor that shapes conversational styles. Chinese culture highly values amicable relationships among family members and tends to avoid direct conflicts in face-to-face conversations (Chan et al., 2009; Luo et al., 2013). Research suggests that arguments with participants’ conflicting opinions may trigger extensive use of reasoning talk (Bova and Arcidiacono, 2014). The observation data showed that most of the existing reasoning talk in this study was in relation to children’s eating issues, where the parents and grandparents may obtain different opinions from the children’s desires.

In terms of reasoning types, the proportion of social reasoning was slightly more than the logical type. This result is consistent with the study that found Australian Chinese mothers used more social than logical reasoning in their daily conversations with their children (Hu and Torr, 2016). However, among the secondary types of social reasoning, the findings indicate that the parents used a considerable proportion of local and coercive reasoning drawn on personal authorities, and in contrast, reasoning talk grounded on cooperative and institutional rules was minor. This finding is different from Hu and Torr (2016) who found the Chinese mothers used more cooperative than coercive grounded reasoning. In this study, most of the instances for the parents to use social reasoning associated with the encouragement for children to eat more and faster, for example, *eat more fish as the bones were all pulled out (local grounding); if you cannot eat faster, then you cannot watch the cartoon show (coercive grounding).* Research suggests that Chinese parents may have high levels of anxiety relating to their children’s physical needs or eating habits (Guo, 2015). The dinner table setting seemed to be a context that triggered the parents’ utterance drawing on coercive power that reflected their anxiety of children’s eating issues. This specific context differs from the situation in Hu and Torr (2016) study where the parent-child conversations occurred in a variety of daily activities (e.g., free play and shared reading) and parents were inclined to use more cooperative reasoning to explain affairs rather than coercive power to direct children’s eating behaviors.

The effect of the communicative contexts on the use of reasoning talk at the family dinner table is noteworthy. Whilst the distribution of logical reasoning was comparatively balanced between contextualized and decontextualized context, most social reasoning was produced in contextualized than decontextualized context. Relevant research suggests that adults tended to apply more logical reasoning in decontextualized context rather than contextualized communication (Hu and Torr, 2016; Hu et al., 2019a), yet much logical reasoning was applied in the contextualized context in this study. The discourse analysis revealed that most logical reasoning is in relation to the description of the attributes of food or human being’s physical that was used by the parents and the grandparents for convincing children to eat more food, for example, *You need to have different vegetable as they have different vitamin*, or *If you don’t eat now, you will feel hungry in the afternoon.* It seemed that the dinner table context provoked parents’ use of both social and logical reasoning for managing the children’s eating behaviors. However, in such a situation, parents’ logical suggestion might also encounter children’s resistance because this language is still directive, which is different from logical reasoning in decontextualized context for explaining affairs in the physical world that is informative.

Social reasoning in this study including local and coercive grounding was more likely to occur in contextualized than decontextualized situations. The interaction effects between family members and communicative context on locally grounded reasoning confirm that both parents and grandparents tended to use this type of reasoning in a contextualized context. Research suggests that local and coercive reasoning involves the manipulation of personal power that has a negative influence on social relationships and may trigger children’s rebellious behaviors (Hasan, 2009). This finding is in line with a large body of research that confirms the pedagogical values embedded in decontextualized communication rather than contextualized communication (Beals and De Temple, 1993; Hu and Torr, 2016; Sheng et al., 2021). In decontextualized context, the family members shifted away from the topics of food or the participants’ behaviors and reasoned about the affairs of “there and then,” for example, when a mother mentioned how to cook octopus from her past experiences, the other family members extended the topic with logical reasons, such as “*adding sugar when it is salty as sugar can balance the savory taste.*” This kind of conversation provided children with opportunities for understanding cause and effect relationships in the physical world.

### The Characteristics of the Reasoning Talk Among the Three Generations

One of the major objectives of this study is to reveal the characteristics of reasoning talk among the three generations of Chinese family members. Basically, the mothers generated the most reasoning talk in both logical and social reasoning. This finding resonates with the research that identifies the dominating role of mothers in family mealtime talk (Blum-Kulka, 1994; Davidson and Snow, 1996). It is also consistent with the Chinese family research that reveals the importance of mothers’ role in children’s home experiences (Luo et al., 2013). Our study further confirms that the significance of mothers’ position in family communications remained in co-parenting situations as well. The fathers in this study were much less active than the mothers. This result is surprising considering the same high level of the educational background of the fathers and the mothers. The disparity between the mothers and the fathers is partially resonated with Li (2020) who reviewed Chinese fathers’ research in the past decades and found the gap between Chinese mother-child and father-child interactions at home. Though the young generation of Chinese fathers has established warmer relationships with children than previous generations, the time they spend interacting with children is much less than mothers, and the gap is increasing. Li (2020) argued that the traditional Chinese fathers’ role as a “breadwinner” rather than a child caregiver at home may foreground this situation. Our study further confirms this statement by identifying the gap between the mothers and the fathers who are at the same educational level (university-qualified). Chinese fathers’ fewer interactions with children than mothers appear generally in daily family life, which is not restricted in mealtime (Du et al., 2018).

The grandparents displayed a low profile at the dinner table as they generated the least reasoning talk in all types. The result of the grandparents playing a subordinate role in the use of reasoning talk indicates that grandparents’ influence on the children’s daily learning experiences at the dinner table is limited. According to previous research, the intergenerational differences in parenting patterns and values between grandparents and parents were in evidence (Ruiz and Silverstein, 2007; Xu and Pei, 2012). Grandparents tended to fall in with children’s feelings and views, or in other words, spoil children, which may result in less use of reasoning talk to explain or argue with children in the mealtime routines. Another reason relates to their educational levels that are generally lower than the parents who were mostly university qualified. Research suggests that speakers’ educational background determines the prevalence and the features of their reasoning talk, and university-qualified adults tend to use more reasoning talk when interacting with children (Hasan, 2009; Hu et al., 2019a). The grandparents were in a “disadvantaged” situation at the dinner table when interacting across the generations.

It is worth noting that although many study results showed the significant role of grandparents’ participation in child-rearing and development, little is known about how it can be specifically explained to indicate the precise meaning of this unique rearing pattern under the condition of co-parenting families. Our study is one of the early endeavors to identify the different influences between parents and grandparents on children’s learning opportunities in daily life with the co-parenting pattern. Given the popularity of grandparents’ involvement in Chinese family’s childcare affairs, future studies are needed to focus on not only parents’ or grandparents’ individual rearing practices but also their shared parenting in the same family, to reveal specific pedagogy around the co-parenting educational environment in families.

Finally, an important result of this study relates to the children’s performance. The preschool-aged children engaged in the dinner table talk actively and they ranked second among the family members in terms of total language messages and their proportion of reasoning talk was close to their fathers’. This result validates the assertion that children could be an important contributor to the family’s mealtime talk (Arcidiacono and Bova, 2015; Sheng et al., 2021). Whilst children are significantly impacted by home language experiences shaped by their parents, they are the creator of the micro-environment as well. Future research undertaking from children’s perspective would further reveal more details about the dynamic conversational picture of family mealtime talk.

## Limitations and Implications

Though obtaining extensive meaningful findings, this study is subject to a few weaknesses. Firstly, the findings cannot be generalized for all families in mainland China, because this study mainly considered the middle-classed families in one small city. Secondly, the language corpus analysis was based on the observational data collected in a way of a snapshot during one mealtime event (video record), and the results may not represent the complete feature of the reasoning talk in these families. Moreover, this study only analyzed the reasoning messages without including the responsive messages generated by the family members. In terms of the completed picture of family reasoning talk, it is meaningful to include the responses to the reasoning messages to further reveal the influences of the different reasoning types.

With the consideration of the above limitations, the findings of this study enhance our understanding of reasoning discourses and mealtime talk in Chinese families. It significantly contributes to the research of Chinese children’s home learning experiences. As one of the pioneering studies that address the Chinese home environment in the co-parenting background, this study provides a case for the research avenue of investigating Chinese children’s learning opportunities through the analysis on the language corpus created by the three generations together, which is different from relevant studies that mainly used the method of survey, interview or direct observation for the exploration of the parents’ child-rearing attitudes and practices. This study affords solid evidence to reinforce the important role of mothers in creating a family learning environment for children. Though Chinese grandparents take considerable responsibilities in childcare duties under the popular pattern of co-parenting, they may not effectively contribute to children’s learning opportunities in the family’s daily life. Parents, and especially mothers, are still the main character and contributors to home learning environments for young children. Finally, the results highlight the pedagogical implication of communicative contexts. We recommend adults pay attention to the variation of communicative contexts in their interactions with children during mealtime. Instead of focusing on the constrictive eating issues, parents’ initiation of topics relating to past and future events or general social issues beyond the dinner table could promote learning opportunities for children who may enjoy meals while engaging in pleasant logical reasoning talk with other family members.

## Data Availability Statement

The raw data supporting the conclusions of this article will be made available by the authors, without undue reservation.

## Ethics Statement

The studies involving human participants were reviewed and approved by Human Ethics Committee of Zhejiang Normal University. Written informed consent to participate in this study was provided by the participants’ legal guardian/next of kin.

## Author Contributions

LL and FZ formulated the study concept and designed the family observation. YH drafted the first manuscript and finally conducted critical revisions. Data collection and analysis were performed by JH and LS. JH provided conceptual commentary and results interpretation. All authors contributed to the article and approved the submitted version.

## Conflict of Interest

The authors declare that the research was conducted in the absence of any commercial or financial relationships that could be construed as a potential conflict of interest.

## Publisher’s Note

All claims expressed in this article are solely those of the authors and do not necessarily represent those of their affiliated organizations, or those of the publisher, the editors and the reviewers. Any product that may be evaluated in this article, or claim that may be made by its manufacturer, is not guaranteed or endorsed by the publisher.
